# Quarterly Summary

**Published:** 1854-01

**Authors:** 


### QUARTERLY SUMMARY.

### DENTAL SCIENCE.

1.—*Dental Patents.*—Professor Townsend's Essay on Dental Patents, read before the American Society of Dental Surgeons at their annual meeting in August last, was published in the October No. of the Dental News Letter.

After quoting from Bouvier's Law Dictionary the definition of a patent, Prof. T. goes on to review the law of patents, and to show that its main object is to induce the ingenious man to make public whatever invention or discovery he claims as his, and which is calculated to promote the progress of science and the useful arts, by protecting his right to the exclusive benefits which may result therefrom for a period of fourteen or twenty-one years. He regards the law as looking to the justice due to the public *as* carefully as to the rights of particular individuals, and that the monopoly is granted for such period of time as it is supposed may operate as an inducement to reveal the secret at once for general use. He points out the manifest injustice of a perpetual monopoly, which would deprive any subsequent discoverer of the same thing from enjoying the benefit of his equal ingenuity and labor. He thinks fourteen years is quite as much as any man is likely to anticipate the invention of other men engaged in similar pursuits. There is, indeed, nothing more common than for the very greatest discoveries to be made almost simultaneously by a number of persons in different parts of the world, or under circumstances which preclude the idea that either is indebted for the hint to another. He considers patent-rights a mere matter of policy, and that no man has any such right, in natural justice and reason, to the monopoly which it confers upon him, and that it properly applies only to those combined results of *knowledge and skill* which produce physical, material, or mechanical results.

Copy-right and patent-right do not stand at all upon the same ground. Although the author is protected by copy-right for a certain time, it does not, in the nature of things it cannot, forbid any other use or employment of the ideas and language of the book, except the tradesman's profit in their republication, in form and substance as they came from the pen of their author, and whatever is essential is subject to remoulding, recombination, reapplication, etc. It is not so with levers, springs, wheels and pullies, and the compositions of chemical elements which patent-rights protect. Coyy-right is not a conflict of interests between an author and the profession for which he labors; but the patentee stands before the world a monopolist of all the value that there is in his invention.

The professor takes the ground that dental patents, applying as they do to the mechanical department of our art almost exclusively, lie wholly within the sphere, and are governed by the laws of *patent-right*, and have no pretension to rank with the principles and subjects of our science, properly distinguished. *As such*, they are not forbidden, either by civil law or individual duty to the mere inventor.

The patentee has no interests in common with the profession, and not being bound by reciprocities of respect and duty, he may hold his wares at *such* price, and I may purchase them on the same ground that I would any other advantageous contrivance, etc., but no idea, principle, or feeling of the profession, and its relations, may justly mingle in the business.

Instead of coming as a brother, the patentee shelters himself behind a piece of parchment furnished by the United States government, warning each and every person against trespassing upon his patented rights. Such persons throw off the character of the dentist and assume that of the mere dealer in wares.

Not so, however, with the author who has secured a copy-right. His ideas are intended to be used by the profession, and he is always gratified in receiving useful hints from his brethren.

"Thus patent-right and copy-right are broadly different in all their bearings upon the interest of the profession, and upon the relations of the practitioners of dentistry."

The faculties of medicine and surgery are united in their opposition to patent medicines, and the American Medical Association declares, that "it is derogatory to the dignity of the profession for a physician to *hold* a patent for any surgical instrument or medicine, or dispense a *secret nostrum*, whether it be the composition, or exclusive property of himself or others. For, if such nostrum be of real efficacy, any concealment regarding it is inconsistent with beneficence and *professional liberality;* and if mystery alone give it value and importance, such craft implies either disgraceful ignorance or fraudulent avarice."

Ours is a legitimate branch of the healing art, and dental patents should not be allowed among us. They have been condemned by the "Mississippi Valley Association," the "Pennsylvania State Society of Dentists," and by the prominent members of the profession generally. It is the duty of members of the profession to lend and give, as well as to borrow and receive.

If from labor, thought, or good luck, a member of the profession has made an important discovery or improvement, and wants the profit of it, he should publish under a copy-right, not take out a patent. But few really great discoverers have ever been able to make anything for themselves out of their special privileges, while "hucksters and pedlars have fattened on their ingenuity."

As the consulting physician imparts his knowledge freely to his professional brother for the benefit of the patient, so is the dentist in honor bound to do the same. The vender who sells a secret, and he who buys, both put themselves out of the profession.

"All dental associations, colleges and practitioners, who have the responsibilities of the profession upon them, and enjoy any of its benefits and honors, should unhesitatingly put dental patents under ban, and treat them as they properly do all other monopolies in the industrial and liberal arts, as things outside of the pale of the fraternity."

2.—*Treatment of Exposed Dental Pulp preparatory to Filling.*—(Continued from page 123, Oct. No. of Journal.)—Professor White, in speaking of the various means which have been adopted for the treatment of the pulp when exposed, says, that his experience in the treatment recommended by many dentists in the use of astringents and capping, has been the same as that of Professor Harris, viz. that only about one case in four has proved successful, and at least as many would be saved without any previous preparation. Mr. Maury's success in the use of the cautery he attributes to the fact that the nerve is destroyed, and most of it removed by the instrument which is passed into the cavity, and that when the nerve is removed far down into the cavity, the bleeding will cease in a few minutes, or hours at most, and this may be as effectually accomplished by a *cold* as a hot instrument, for in either case the pulp is destroyed. Where it can be properly applied, he considers the *actual* cautery the best means of destroying the pulp, particularly in the roots of the front teeth where one application will be sufficient; but he does not use it now excepting in the preparation of those roots for pivot teeth. Care is required in using it that it does not come in contact with the parietes of the internal cavity, or retained in it unnecessarily, or the heat caused thereby may produce inflammation of the alveolo-dental membrane, followed by abscess. Acute pain may be the result if the pulp be but partially destroyed; but when entirely, the pain immediately ceases.

Stimulants, as recommended by Mr. Bell, who is opposed to the use of the actual cautery and corrosive acids, the professor is not prepared to adopt. If their continual application will but gradually, as is asserted, wear away the sensibility of the membrane, their effect is as uncertain as the application of astringents; but if they cause absorption of the whole pulp, they succeed upon the same principle as that of the cautery, properly applied. Formerly, Prof. W. was in the habit of inserting a small gold tube in his fillings to give free exit to the accumulated fluids in the pulp cavity, upon the same principle that some now perforate the neck of a tooth with a small drill; but having discovered that he could destroy the pulp without rendering the tooth a useless foreign body, he has abandoned it.

*The Concentrated Acids.*—Of these, arsenious acid is most commonly used, and the professor thinks it the best agent when properly combined with other substances, as it can be applied with the same facility to the back, as to the front teeth. When applied by itself, it does not necessarily destroy the vitality of the nerve, but produces intense pain and acute inflammation by being taken up by the absorbents, requiring the immediate removal of the tooth, and consequently its use has been opposed by the best dentists. Whatever portion of the pulp is destroyed must be removed, otherwise it will cause inflammation.

*Arsenious acid, kreasote and morphia* is, perhaps, the best form of using arsenic.

℞.—Arsenious acid, gr. xxx.
Morphia sulphas, gr. xx.
Kreasote, 2 S.
Misce.

"Put the arsenious acid and kreasote in a glazed mortar, and grind them till the arsenic becomes impalpable, (adding kreasote to keep the mass of about the consistency of cream,) then add the sulphate of morphia, and mix it well, still adding kreasote; it will dissolve in the paste. Prepared in this way, the arsenic is in better condition to unite speedily with the pulp than the mere dry powder of arsenic, on account of the kreasote holding a large quantity of it in solution, and it becomes more minutely divided. Great care must be taken to cleanse out the external cavity of the tooth, so as to place the paste in immediate contact with the pulp. A pledget of cotton about the size of a small pin's head, steeped in the paste, is sufficient. If the pulp bleed when the cavity is cleansed, wait until the bleeding subsides before applying the paste; the cavity may then be filled with cotton, and left in from ten to twenty hours." In a young patient, remove the paste in much less time.

This form of using arsenic destroys the pulp in less time, with less pain, and less inflammation of the external membranes than when applied alone, and causes a more extensive and perfect slough of the pulp, which can be removed far down in the roots, on which depends very much the success and permanency of the operation. The pulp having been removed, a period of from three to six days is usually permitted to elapse before plugging it, in order to remove the clot of blood which may be formed, and otherwise securing a proper condition of the cavity, which should, in the mean time, be stopped up with cotton, and not used in mastication. In one hundred cases treated in this manner in 1842, but sixteen gave pain, averaging for each one hour. Six of the whole number have since been extracted on account of alveolar abscess. The paste should never be applied in the teeth of a patient, where, from the age, we have reason to think that the roots are not yet fully developed.

3.—*Deposition of Ivory in the Wounded Tusk of an Elephant.*—W. D. Porter, U. S. N., and Surgeon Dentist, in a letter published in the Dental News Letter for October, states that there is in the possession of S. G. Willing, of New Rochelle, New York, the tusk of an elephant, wounded by an iron ball of an half ounce weight, and a half inch in circumference, the ball passing an inch and a half through the enamel and ivory, through the nerve cavity, and lodging on its opposite side in the ivory. Nature has performed a cure by depositing bone or ivory. The ball shows a black oxyd, and that portion of it exposed in the nerve cavity has an ivory covering.

Mr. Porter had caries removed from one of his molars fifteen years ago, and the cavity being very narrow, he ordered a long incision to be made and left without a filling, and had the dens sapientiæ removed in order to test the renovation of both ivory and cementum, and the result is that the tooth has entirely recovered. The above facts remove all doubt from his mind as to the renewal of both the ivory and enamel of the teeth under certain circumstances.

4.—*Freak of Nature.*—J. F. Terry, of Tecumseh, Michigan, in a note addressed to the Dental News Letter, says, that he had just examined the mouth of a girl six years of age, in which there are six perfect incisors in the inferior maxillary of the temporary set, with cuspidati and molars as usual.

5.—*The Insertion of Teeth upon Platinum Pivots.*—In an article from Frank Fuller, of Portsmouth, New Hampshire, published in the Dental News Letter for October, he recommends the following mode of inserting pivot teeth, viz.

"Having selected the tooth, and adapted it, by grinding, to its place of destination, I take a piece of platinum wire, about one-third smaller than the pivot-hole in the tooth selected, and file upon its surface, at one end, a few irregular notches; inserting the notched end of the wire in the tooth, and placing it in the centre, or at either side of the cavity, according to the necessities of the case, I proceed to pack around it, by the aid of proper plugging instruments, as large a quantity of jeweller's "soft enamel" as can be introduced. This being done, I imbed the tooth, with its cutting surface down, in plaster, to which has been added a quantity of sand or asbestos, leaving only the pivot and the orifice of the pivot-hole, with its contents exposed. Heating the job in the furnace, to redness, to expedite the process, I remove and apply the blow-pipe, until fusion of the enamel occurs. The tooth, when cooled, will be found to be firmly connected to the pivot by the intervening enamel, and will resist every force which may be applied for its removal. I proceed to cut the pivot of a proper length, file to a square form, and point it, and cut from the butt of the tooth towards the point of the pivot, numerous barbs, to retain it more firmly in its place. I then fit a piece of some close wood—of which I prefer the locust—to the cavity previously formed in the root, in the usual manner, push the pivot to its place, and have a firm, substantial, and elegant piece of work.

"The pivot can be filled to any shape, and bent as may be desired, and thus an accurate adaptation of the tooth to any case is insured. I should have preferred, however, the gold to the platinum, for various reasons, were it not for its liability of fusion from the very considerable heat employed. The soft enamel can be obtained of some of the watch-makers in our larger cities, or by pulverizing the enamel of an old watch-face."

6.—*Caries of the Teeth.*—Prof. J. Taylor's answer to Dr. Drake's interrogatories, was continued in the October No. of the Dental Register. Dr. T. proceeds to consider the question, "What is the effect of repeated salivations on the teeth and gums?" Mercury in its pure state has no affinity for the teeth. Analysis proves this, and teeth immersed in this substance for months show no signs of decomposition. This fact being established, the numerous cases of tooth-ache which occur after an attack of sickness, and which are so often attributed to mercury, must depend upon other causes rather than the direct chemical action of this medicine. The most reasonable conclusion is, that such teeth have been decaying for some time, but during healthy operation of the economy, no just cause for derangement has occurred, and hence no pain—but the indisposition which rendered it necessary to take medicine, has vitiated the secretions of the mouth. Acting as irritants, the teeth thus affected, they result in tooth-ache. An analysis of the saliva of a patient laboring under mercurial ptyalism seldom shows an acid reaction; but teeth already decaying may have the disease accelerated by such condition of the secretions, although sound teeth would not be more affected thereby than under any other derangement of the healthy condition of the mouth. In many cases where persons have been badly salivated two or three times, the teeth remain perfect. The injurious effect of salivation upon these organs during the period of formation, causing diseased action, which interferes with their development, has already been referred to.

The injury to gums from repeated salivation is not doubted. It causes inflammation and swelling of the gums, thickening of the periosteum of the teeth, teeth raised in their sockets and loosened, separation of the gums from the necks of the teeth, thickening of the alveolo-dental membrane, absorption of the alveolar processes, and wasting of the gums. One or all of these conditions may be produced.

The opinion expressed by Prof. Bond, in his work on dental medicine, in regard to the non-injurious effects of mercury on the teeth is quoted as satisfactory.

What are the effects of tobacco on the teeth, and are those of chewing and smoking the same?

In its pure state, the use of tobacco is not injurious—excepting where a constitutional diathesis and a predisposition to decay exist—but preservative, the friction produced by chewing it keeping the teeth cleansed. While the friction abrades teeth, it also preserves them, as those teeth used most in masticating our food are always the soundest.

Prof. T. is opposed to the use of tobacco, and believes it to be injurious to the constitution generally, and the gums may suffer from the long continued pressure of the quid. A free use of it for a long time discolors teeth, presenting a disgusting appearance.

*Smoking* is considered more injurious than chewing, producing a debilitating effect upon the gums, and it is probable that in many instances serious mischief is caused by it.

*Tartar.*—This subject is treated at considerable length. Prof. T. thinks it probable that it is primarily obtained from the saliva as it contains nearly if not all the elements of tartar. It has been found in the ducts which pour out the saliva, and it is usually deposited on the superior molars and the lingual surface of the inferior incisors opposite to the salivary ducts. If it were a deposit from the mucous secretions, it wouid be as likely to be deposited first about those teeth farthest removed from the salivary ducts as opposite to them. Delabarre's theory that tartar is caused by an exhalation from the mucous membrane of the mouth when in a state of inflammation, is not tenable. When inflamed, the mucous membrane throws out an acid secretion which gives no deposition of tartar. This acid state of the mouth is one great cause of the decay of teeth; but those most disposed to decay, are less disposed to accumulate tartar.

It is not thought necessary to allude specially to the effects of this substance on the teeth and gums, or the different effects resulting from different temperaments.

7.—*Clasps.*—Dr. B. T. Whitney, of Buffalo, N. Y., has an article in the Dental Register, for October, on the adaptation of clasps so as to prevent the loss of, or do the least injury to, teeth.

The attaching a small plate by making the bearing simply on the palatal side of the remaining teeth, although admissible, is liable to serious objections, particularly on account of the pressure being made upon the part most liable to injury from clasps, and unless very nicely adapted, the principal bearing being around the neck of the tooth, or above the central and rounded part of the crown, it will not only be too easily displaced, but is also likely to force the natural teeth outward. The exercise of good judgment in the selection of a tooth to be used for the support of the plate—without regard to the expense of the plate, or labor in fitting—will make it seldom necessary to use the file, and will give firmness to the piece.

The point most liable to injury by clasps is on the lingual surface, the labial, where the ends of the clasps are some distance apart, being free from decay or injury.

Dr. W. is in the habit of leaving as much of the tooth exposed to its native element as possible, the cleansing effects of the saliva, tongue, &c., which can be accomplished without weakening the clasps, or the stability of the plate. The strip of gold used for the clasp is as wide as the length of the crown will receive, and in length so as to embrace only so much as is really necessary for the support of the piece. "Fit the plate intended for the base or support of the artificial teeth accurately to the neek of the tooth, so as to encircle the lingual half, or as nearly as possible, but leave a space of from one to two lines between them. The clasps is accurately fitted to the tooth, then with the plate—held firmly in its place—mark upon the clasp at the points of the plate at the anterior and posterior approximal sides of the tooth, and cut out a semicircular piece from the clasp, between these edges, leaving but a small portion to come in contact with the plate at each point, just enough to solder them firmly together." Then solder at one point only before placing the piece in the mouth to see if the position of the clasp is right, and giving an opportunity of making any desirable adjustment. This leaves the lingual side of the tooth most liable to decay or injury, almost entirely free, and is probably the best mode of avoiding injury.

8.—*Tooth-drawing.*—Dr. C. T. Cushman publishes in the Dental Register, some difficult cases of extraction of teeth. One was that of a man-servant who had been under treatment for months for what his physician termed an "unmanageable head-ache," and finally suspecting that the left inferior dens sap., which was much decayed, was the cause of the pain in the head, attempted to extract it, but broke it off below the level of the gum. Dr. C. tried Harris' forcèps, slender root forceps, key and elevators, without success. The punch was then resorted to, and the *outer* side being the most prominent portion of the fractured roots, he covered the fingers of the left hand with a napkin, placed them within the mouth, clasped the left side of the inferior jaw, while the thumb placed along the margin of the gum served as a fulcrum. The punch, firmly grasped in the right hand, was then forced down as low as possible between the gum and the root on the outside, the handle depressed, then rising and pushing it toward the centre of the mouth, the roots instantly gave way. The roots were long, and periosteum extensively ulcerated, doubtless causing the long continued head-ache.

By this method of thus using the punch, Dr. C. has rarely failed in such cases, and never found it necessary to cut away the alveolus.

Case VIII was *"a stumper for the dentist."* This was an inferior *dens sap.*, growing in such a position as to be completely dove-tailed behind the second molar. Two persons had tried to extract this tooth without success. The tooth was firm, and no attempt was made by Dr. C. to extract it, and thinks that the *Physic forceps* would be the best instrument in such a case. The tooth would have to be forced back at least a line to clear the second molar.*

9.—*Practice of Dentistry.—A few suggestions to young men commencing, or about to commence the Practice of Dentistry,* by J. F. JOHNSTON, D. D. S , of Indianapolis, Ind., published in the last number of the Dental Register. We heartily approve these suggestions which, though oft repeated, are, unfortunately, always seasonable.

The doctor speaks of the difficulties young men have to contend with in the commencement of practice, and warns them against being sanguine that those difficulties will cease as soon as the first obstacles are surmounted. The cases to be treated both in surgical and mechanical dentistry are so various that there is always something new, and perhaps difficult or perplexing, requiring zeal and untiring energy to treat them successfully, and thus elevate, instead of disgracing the profession. He deplores the fact, that so many who are grossly ignorant of the duties devolving upon the dentist, should rush into the profession, and that others are satisfied to remain indolent instead of becoming working, energetic men. Neither carelessness nor laziness will answer for the respectable dentist. He cautions the young dentist to be *conscientious,* to attempt whatever is practicable, however difficult, to take time to do his work thoroughly—even should it prove tedious to the patient—for by so doing he will in the end secure the confidence of his patients. Those who will not submit to proper treatment are better lost than retained. Do not insert teeth in a mouth full of old roots, even if Dr. —— has offered to insert a first rate set without their removal. Let not the want of funds tempt you into bad practice. The quack may insert the teeth, but the patient will probably return to you

*In a similar case in our practice not long since, we cut freely around the crown, made a free lateral incision, and found no difficulty in removing the tooth with the root-forceps. We seized the tooth in such a manner as to cause it to glide over the posterior surface of the second molar, elevating the crown at the same time that the usual motion was applied. A number of attempts had been made to extract this tooth by a dentist in the country without success.

when they can no longer be endured, with regrets that he did not employ you at first. Success will finally attend you, and you will have the satisfaction of knowing that your patients have entire confidence in you.

In conclusion Dr. J. says, "I have been told of a certain dentist who occasionally preaches for the avowed object of saving the souls of his dear hearers; and after having discharged this christian duty, he very affectionately tells them he will save their teeth. It is supposed by some, that he comes as near saving the former as the latter, and that he saves more of the people's *funds* than either." A dentist not only can, but should be a religious man.

We have heard of a similar case in this region, with the apprehension expressed that if the preaching should prove to be no better than the dentistry, that the hearers would probably find themselves in a sad predicament.

10.—*Supernumerary Dens Sapientiæ.*—Dr. J. Richardson, of Cincinnati, states, in the October No. of the Dental Register, that he had recently extracted both the inferior dens sapientiæ for a gentleman about thirty years of age, each having four fangs, well defined in the left, less so in the right. The crowns were unusually large, and there was the usual complement of teeth in front of them. The gum on the left side was soon restored to health; but not so on the right. The patient returned some days after the latter was extracted, complaining of pain at the bottom of the cavity, soreness about the angle of the jaw, extending into the temple and neck. On a careful examination the grinding surface of an apparently well developed crown of a second wisdom tooth was discovered, occupying a position directly underneath the one extracted.

11.—*Formula for Pain in the Teeth and Jaws.*—C. P. Culver, of New Castle, Ky., in a note to the Dental Register, says that he has found the happiest effects produced by the use of the following

1 oz. hartshorn;
1 oz. chloroform;
2 dr. oil cloves;
2 dr. nitric acid;
2 dr. ex. camphor;
2 dr. ex. nutgall.

Care should be taken to prevent the discoloration of the teeth which may be caused by its use.

The editor of the Register objects to the nitric acid.

### ANATOMY AND PHYSIOLOGY.

12.—*Cell Growth.*—Mr. T. H. Huxley has published a review of Schleiden and Schwann's theory of cells and their uses, in which he takes very decided ground against the commonly received opinions on this subject.

He calls in question, first, the doctrine of the anatomical independence of the cell, which he regards as simply a cavity in the intercellular substance, containing an active nucleus of vegetable tissue. He says it is "a homogeneous, cellulose-yielding, transparent substance containing cavities, in which lie peculiar vesicular bodies, into whose composition much nitrogen enters." For these he proposes new terms. The general mass which contains the cavities, and *which forms their walls*, he calls *periplast*, while the contained vesicular bodies he names *endoplast*. The cavities (commonly known as cells) he maintains to be formed, as to their outer boundaries, of periplast, and so not to be anatomically distinct, but identical with that substance throughout the plasma.

Next to the outer wall of the cell, that is to say, in close contact with the periplast lies the essential, active part of the endoplast, the *primodial utricle.* The nuclei, when present, are identical in chemical composition with this *utricle*. These, with the protoplasm, he regards as subordinate, and almost *accidental* anatomical modifications of that active element.

He denies, *in toto*, Schleiden's description of the relation of the nucleus to the cell-wall, and declares that cell-development goes on by the growth of the periplast around and between the cavities. This growth and development, in his view, are not the result of activity of the cells exclusively, but depend upon the equal extension of the periplast and endoplast.

In examining Schwann's comparison of cartilage with vegetable tissue, he attempts to show that he makes the mistake of comparing the cartilage nucleus with the plant nucleus, whereas the former is really analogous with the primordial utricle of the vegetable. The chondrin wall of the cartilage must then be the analogue of the cellulose wall of the plant, both being periplastic elements.

The periplast he regards as the seat of all the most important changes which take place. By its differentiation, all the changes, whether morphological or chemical, are produced.

Its chemical metamorphoses may be either of *conversion*, as when cellulose is changed into xylogen, or of *deposit* as when silica or calcareous salts are introduced into the tissues.

The structural metamorphoses are classified under the heads of *vacuolation*, or the formation of cavities, and *fibrillation*, or that change which produces a tendency to break up in certain definite lines.

In the development and exposition of these notions, he introduces several facts very much at variance with received doctrines. He describes muscular fibre fading insensibly into tendon, or branching and terminating in stellate bodies belonging to the connecting tissue.

---

13.—*Histolysis.*—Under this head, in the annals of micrology of the *British and Foreign Medical and Chirurgical Review*, Mr. R. B. Lyons has described the morphological changes which the different tissues undergo during the progress of putrefaction. These are but a brief statement of some of the results obtained by him and published in the *Transactions of the Royal Irish Academy*. In that he sums up his results as follows:

"In considering the chief results arrived at in the study of the process of putrefaction, I am led to believe,

"1st. That, concurrently with the first order of chemical changes, a certain order of morphic changes takes place before the final dissolution of organic structures, by the action of chemical and physical force.

"2nd. That this series of changes may, under normal conditions, take place very slowly, so that, at the end of many months, and probably even of much longer periods, we are still enabled, by the microscope, to recognize and identify structures of great delicacy, such as elementary muscular fibre, and that this knowledge admits of important application.

"3rd. That in this process of histolysis, the first changes consist in the softening, disunion, and separation from each other of the morphic constituents of the tissues, each of which is then subjected to a process of disintegration.

"4th. That granules and granular corpuscles appear at an early period, arising, probably, from recombinations of the organic fluids. Animalcules appear at this stage.

"5th. That granules, corpuscles, vesicles, cells and granular masses of various kinds and sizes, may form in fluids and tissues undergoing histolysis, in which no such elements exist when in their normal states.

"6th. That generally, in the progress of histolysis, structures very similar to those which are arranged under the first group, or the aplastic elements of histogenesis, form at different stages, and that they exhibit the same modes of growth and development, but, like them, are incapable of producing higher forms.

"7th. That these morphic elements of histolysis pass gradually into lower forms, exhibiting occasional instances of endogenous fissiparition, granular disintegration, and other changes, and that the cellular and corpuscular elements, by forming media for endosmose and imbibition, may aid in the disintegration of contiguous structures.

"8th. That certain elements may pass directly into a state of molecular disintegration.

"9th. That certain corpuscles, of peculiar characters, and not identical with any known normal elements, are occasionally found.

"10th. That a period arrives, at which chemico-physical forces prevail, which is evidenced by the passage of certain elements into crystalline forms, others passing off by volatilization, solution, &c., and that in this way the final dissolution of a tissue is accomplished, the several morphic changes which take place probably facilitating and preparing the way for the action of the chemical forces.

---

14.—*Development of Blood Globules.*—Moleschott has ascertained, by a series of experiments on frogs, that when the liver is extirpated, the number of white globules in the blood is increased. In the cardiac blood he found 1 white corpuscle to 2.24 colored, the natural proportion in these animals being 1 to 8. In the blood of the liver, the proportion was 1 to 5.88. In the splenic blood there were more white than red corpuscles. *Hence the liver appears to be an organ in which, to a very considerable extent, the conversion of white corpuscles into red takes place.*

When the spleen is extirpated, there is a slight increase in the proportion of colored corpuscles. When both liver and spleen are removed the quantity of colored corpuscles is diminished to one-fourth the normal amount.

15.—*Temperature of the Blood.*—The younger Liebig has been engaged in researches upon the relative temperature of arterial and venous blood. He has found, contrary to the commonly received opinion, that venous blood is always hotter by from .07° to .19° C. than arterial.

In order to get at these results, he killed a dog, and tied a ligature tightly round the neck, to prevent collapse of the lungs. Some air remaining in the cells of these organs and the circulation still continuing for a while, the blood would be acted upon by the air, and arterial blood would always be found in the left side of the heart. The thorax was now opened, by the smallest possible aperture, the aorta tied at its bend and the descending cava close to the auricle; then the dog being raised, the vessels were drawn so as to make the opening in them the highest point. Thermometers then being passed into the two ventricles through these vessels, the opening in the thorax was filled with carded cotton, to prevent the more rapid cooling of the thinner ventricle.

The results are:

1st. *As to the difference of temperature between the two kinds of blood.* In the recently dead animal, the blood of the right ventricle was once hotter and twice equal to that of the left. In the ascending cava, the blood was 0.72° C. warmer than in the carotid. In the *living* animal the venous blood was, as we have said, constantly .07° to .19° C. warmer than the arterial.

2d. *As to the differences of temperature in the venous blood at different parts of the circulation.* The temperature of the blood, in the large vessels coming from the head and upper extremities, rises as it approaches the vena cava inferior, in which it attains the highest temperature. In the vena cava superior this increase is not very rapid, but in the auricle, where this blood is mixed with the abdominal blood, the rise is well marked. A similar rise takes place in the iliac vein. In the extremities the temperature is lower than in the trunk of the venous system. In arterial blood the variations are much smaller, and become altogether inappreciable at less than 6 centimetres from the heart.

3d. *As to the changes of temperature at one and the same level of the arterial or nervous system.* The temperature in the veins varies regularly with the respiration. In the superior cava the rise takes place at the end of inspiration, reaches its highest point in the interval, and the temperature is lowest after expiration.

Maximum. Minimum. Maximum.
Inspiration —— Expiration —— Inspiration —— Expiration.
The oscillations in regular breathing are from 0.07° to 0.19° Centigrade.

Liebig explains these phenomena in this manner: In inspiration, the pressure of the diaphragm and abdominal viscera causes an increased flow of blood from the inferior cava towards the ventricle; while in expiration the flow takes place toward the inferior cava. Now the blood of the inferior cava being warmer than that of the superior, the blood must be hottest just after inspiration.

16.—*Function of the Valves of the Heart.*—Nega has advanced some new views of this subject, founded upon vivisections and experiments on the dead heart. He thinks he has proved that the auriculo-ventricular valves are closed by the sudden contraction of the auricles, after the ventricles are partially filled. Secondly, he has seen, during his vivisections, the tense valvalar flaps drawn down by the action of the papillary muscles, which, according to Kürschner, almost disappear into the substance of the heart, during the systole. Thus, the valve acts not only as a valve to the auricular opening, but as a forcing pump to the ventricle, and a suction pump on the auricular side. The first sound, therefore, he thinks, is caused by the tension of the valves and the tendinous chords, caused by the contraction of the papillary muscles and the hydrostatic pressure of the blood.

17.—*The Phrenic Nerve.*—Luschka has published a memoir on this nerve, in which he arrives at several new conclusions.

It comes off from the cervical plexus, usually from the fourth cervical nerve exclusively, though sometimes it interchanges filaments with the neighboring nerves. In regard to this point, he admits the union with the middle or inner cervical ganglia, but denies that with the pneumo-gastric. The communication with the descendens noni, was only observed in three cases, and then a microscopic examination showed that it was only apparent and not real. A connection, however, of common origin, exists between this nerve and the cutaneous branch of the fourth cervical nerve of the shoulder, which will account for the pain in the right shoulder accompanying hepatitis. This connection is sometimes rendered closer still by arched fibres passing from the phrenic to this same cutaneous nerve.

As it passes over the pericardium, it is occasionally diseased in certain pulmonary disturbances—tubercle, for example. Branches of it are distributed to the pleura, and therefore Luschka thinks that, in pleurodynia, blisters ought to be applied above the clavicle, so that the subsequent narcotic dressing may be applied in the neighborhood of the cutaneous ramification of the nerve.

The lower cava and the right auricle are fed by this nerve in company with the sympathetic experiments, performed on dogs and rabbits by the author, show that stimuli applied to the nerve, produced contractions in the auricle isochronous with those of the diaphragm and not with the contraction of the compartments of the heart.

The phrenic nerve also communicates very freely with the sympathetic and with its fellow of the opposite side, and sends branches to the peritoneum, the liver and supra-renal capsules. Some of the branches are directed towards the navel, which accounts for the fact that, in periteneal inflammation, the pain often begins in the region of the navel. From the reflex action of these nerves, in all probability, arises the vomiting which attends peritonitis.

The phrenic nerve, therefore, is both motor and sensory, and besides the functions already adverted to, it brings about a double interchange between the sympathetic and spinal nerves. Luschka seems to think that it is the special communication between these two great classes of nerves.

---

18.—*Influence of the Pneumo-Gastric Nerves in Digestion.*—Bidder & Schmidt, whose contributions to the Chemistry of Digestion we noticed at length in our last number, have also added to our knowledge of the physiological relations of this fundamental function of life. They found that:

1. Section of the pneumo-gastric nerves did not diminish the sensation of hunger, and increased that of thirst. The œsophagus being paralyzed in its lower part, the food or saliva could not pass into the stomach and was again rejected. The absence of the usual absorption of fluid and saliva by the blood vessels, explained the thirst.

2. The motions of the stomach were not impeded. Food introduced into the fistula was forwarded in the usual way, and was not returned by the vomiting which was going on. Thus the vagus, in the neck, does not possess those fibres by which the muscular coat of the stomach is connected with the centre of its regular actions. Nevertheless, irritation of the vagus in the neck excites the action of the stomach.

3. The secretion of the gastric juice was *little diminished.* When any diminution took place, it was to be attributed to the lack of the stimulus of food in consequence of the œsophageal paralysis. By reason of the continued evacuations of the animal, the necessary water for the preparation of the gastric secretion was not to be had, so that the elimination of that fluid was considerably diminished, but it was restored again as soon as the alimentary canal was sufficiently moistened. The gastric juice formed under a general deficiency of fluid was far less acid than that furnished after a new supply of water. This, however, which in one case was observed to rise continually till the time of death, differed little from the reaction before the operation, being, on an average, neutralized by .413 per cent. of potash. Thus the *chemical constitution of the gastric fluid* is not remarkably altered by section of the vagi.

4. The *quantity of albumen digested is materially diminished* by section of the vagi, though the function is not destroyed; an effect which, it would appear, must be attributed to the alteration in the quantity of the secretion, the only known change in the action of the stomach caused by the operation.

It will be observed that these results are the very opposite of those of Bernard, who stated that, after section of the vagi in the middle of the neck, the motions of the stomach ceased, the secretion of gastric juice was instantly arrested, and in no case did any part of the food go through the peculiar changes of chymification.

19.—*Relations of Electricity to Secretion.*—Mr. Baxter, who has been engaged in experiments upon this subject, has found that when a circuit is established in a gland, while the process of secretion is going on, the two fluids, the blood and the secretion are in different electric states, the former being positive, the latter negative.

During respiration the same change is observed. The arterial blood is positive, the venous negative.

20.—*Structure of Cartilage, Bone, Dentine, &c.*—Dr. Hoppe has published in *Virchow's Archiv*, the result of some investigations, which he has recently made by the aid of Papin's digester. He finds that the cells resist the action of the boiling water with such energy that he is compelled to believe them to possess non-gelatinous walls of their own, and not to be, as some have supposed, mere cavities in the tissue.

By boiling in Papin's digester, the intercellular substances of cartilage could be dissolved, and the cells were found floating free in the liquid. Elastic fibro-cartilage gave the same result, disproving Mülder's dictum that "the cells of fibro-cartilage yield chondrin on being boiled."

Bone, from which the earthy matter was removed by a dilute acid, dissolved entirely with the exception of the lacunæ and canaliculi, showing that these structures possess true non-gelatinous walls. Dentine gave analogous results, and Hoppe concludes that the tubes and fibrillæ of that structure possess walls of their own which contain no chondrin.

He arranges the tissues of the vertebrata in two classes: 1. Those which consist of cells and intercellular substance, as areolar tissue, cartilage, fibro-cartilage, bone and tooth: 2. Those which consist of cells alone, as epithelium, muscle, nervous and elastic tissues. This distinction is believed to hold chemically, anatomically and physiologically; since the first class comprises the *supports* of organs, the second, the organs themselves.

21.—*Capillaries of the Liver.*—It will be remembered that some doubt has existed in the minds of physiologists in regard to the capillaries of the liver, whether the minutest of them had a true tunic, or whether they were only channels in the proper substance of the liver. Mr. Rainey, in a communication to the *Journal of Microscopical Science*, states that he has shown them to be, like all other capillaries, possessed of a true tunic, and that they are about 1-3000

of an inch in diameter, inosculating and leaving meshes about 1-1000 of an inch in diameter. He thinks the reason this tunic is so difficult to demonstrate, is, that it is so thin and delicate, and adheres so firmly to the hepatic corpuscles, no basement membrane having been demonstrated in the liver.

### EDITORIAL DEPARTMENT.

